# Quantification of the Iodine Content of Perigastric Adipose Tissue by Dual-Energy CT: A Novel Method for Preoperative Diagnosis of T4-Stage Gastric Cancer

**DOI:** 10.1371/journal.pone.0136871

**Published:** 2015-09-15

**Authors:** Li Yang, Gaofeng Shi, Tao Zhou, Yang Li, Yong Li

**Affiliations:** 1 Department of CT, The fourth Hospital of Hebei Medical University, Shijiazhuang, Hebei Province, China; 2 Department of surgery, The fourth Hospital of Hebei Medical University, Shijiazhuang, Hebei Province, China; Duke Cancer Institute, UNITED STATES

## Abstract

This study investigated the utility of quantifying iodine concentration (IC) in perigastric adipose tissue, using dual-energy computed tomography (DECT), for the detection of T4a-stage gastric cancer. Fifty-four patients with gastric cancer were enrolled at the Fourth Hospital of Hebei Medical University between January and June 2013. Patients were imaged preoperatively with conventional computed tomography (CT) scans and DECT, and the IC in perigastric fat adjacent to the tumor calculated from arterial phase (AP) and portal venous phase (PVP) images. The patients subsequently received surgical treatment (gastrectomy), and histologic analysis of resected specimens was used as a ‘gold standard’ reference for cancer staging. Receiver operating characteristic (ROC) curve analysis was employed to assess the utility of DECT for identifying T4a-stage gastric cancer, with optimal IC thresholds determined from the area under the ROC curve (AUC). Postoperative histology revealed that 32 patients had serosal invasion (group A), and 22 did not (group B). The accuracy of conventional CT for distinguishing stage T4 from non-T4 stages was 68.5% (37/54). IC was significantly higher in group A than in group B (AP: 0.60±0.34 *vs*. 0.09±0.19 mg/mL, p<0.001; PVP: 0.83±0.41 *vs*. 0.27±0.21 mg/mL, p<0.001). The sensitivity, specificity and AUC for detecting serosal invasion were 77.1%, 79.2% and 0.89 at an IC threshold of 0.25 mg/mL for AP images; and 80.0%, 79.2% and 0.90 at an IC threshold of 0.45 mg/mL for PVP images. These results indicated that Iodine quantification in perigastric fat using DECT is an accurate method for detecting serosal invasion by gastric cancer.

## Introduction

Gastric cancer is one of the most frequently diagnosed cancers, and is a leading cause of cancer-related deaths worldwide [1–3]. The preoperative staging of gastric cancer is widely recognized as an invaluable aid for determining the optimal therapy and evaluating tumor resectability and patient prognosis [4–6]. The TNM system is commonly used to stage gastric cancer, with T4 defined as a tumor that invades the serosa [3]. Accurately differentiating T4a-stage gastric cancer from T3 or earlier stages is particularly important with regard to preoperative selection of appropriate treatment strategies, including the requirement for multi-organ surgery [7–9]. Neoadjuvant chemotherapy is strongly recommended for patients with T4 staging and lymph node metastasis, and may be beneficial to those with T4a stage in down grading the tumor prior to resection allowing in some cases curative resection [10]. Multi-detector computed tomography (MDCT) is often chosen as the modality for preoperative staging, and has been shown to have an overall accuracy that approaches 90% [5,7,9]. Nonetheless, preoperative staging with MDCT can be difficult because the serosal surface is very rough and the adjacent adipose tissues are generally turbid, so increased density could reflect several different phenomenon including tumor invasion and reactive fibrous connective tissue hyperplasia; therefore, the specificity of MDCT is relatively low. As MDCT does not show complete agreement with postoperative staging by histologic analysis of surgically resected specimens new approaches are needed to improve the sensitivity, specificity and accuracy of imaging modalities for the preoperative staging of gastric cancer.

It has been demonstrated that dual-energy CT, including dual-source dual-energy CT (DECT) is capable of quantifying the iodine concentration (IC) in tissues *in vivo*. [4] Therefore, DECT could potentially be used to measure the iodine content of tumor-invaded perigastric adipose tissue of the lesser and greater omentum, and this may represent a novel approach to more accurately detect T4a-stage gastric cancer. We hypothesized that quantification of the iodine concentration in perigastric adipose tissue using DECT could help to distinguish T4a-stage gastric cancer from earlier stage tumors. Therefore, the aim of the present study was to investigate the relationship between the iodine concentration in perigastric fat, measured using DECT, and T4a-stage gastric cancer, and to determine the sensitivity, specificity and accuracy of DECT for identifying T4a-stage gastric cancer, using histologic assessment of surgically resected specimens as the ‘gold standard’ reference for tumor staging.

## Materials and Methods

### Patients

This was a cross-sectional diagnostic study that enrolled consecutive patients with gastric cancer confirmed by endoscopic biopsy, who were referred between January 2013 and June 2013 to the Department of CT, The Fourth Hospital of Hebei Medical University, Shijiazhuang, China, for preoperative CT scanning to stage the disease and assist with treatment planning. Patients were excluded from the study if: surgical resection of the gastric tumor (gastrectomy) was carried out more than 1 week after the CT scan; the patient was allergic to contrast medium; or the patient had T4b stage cancer that was easily diagnosed by CT to have invaded other organs. All the included patients underwent a three-phase CT scan: pre-contrast single-energy CT imaging, and contrast-enhanced DECT imaging at arterial and venous phases. Some patients had too thin fat layers for CT imaging and were considered to have technique failure. Histologic examination of resected specimens was performed in a blinded manner after surgery, and served as the ‘gold standard’ reference for tumor staging. Two senior radiologists, who did not know the endoscopic findings or pathological results, were assigned to reconstruct the merge images during the venous phase (thickness of 1.5 mm, using the B30 algorithm), analyze their axial views and multi-planar reformation (MPR) images and discuss the tumor stages. Criteria for tumor staging were based on the TNM staging system for gastric carcinoma (7th edition) by the American Joint Committee on Cancer [3]. According to the postoperative pathology results, the included patients were assigned to one of two groups: group A, serosal invasion (stage T4a); or group B, intact serosa (stage T1–T3).

The study was approved by Institutional Ethics Committee of The Fourth Hospital of Hebei Medical University, and written informed consent was obtained from each patient prior to inclusion.

### Image acquisition

All CT images were acquired with a dual-source dual-energy CT scanner (SOMATOM Definition Flash; Siemens Healthcare, Germany). Each patient was instructed to fast for at least 6 hours before the CT examination. Ten minutes before scanning, each patient was administered 10 mg anisodamine intramuscularly (to reduce the tension of the gastrointestinal tract), and drank 800–1000 mL water (to fully expand the stomach). The pre-contrast images were acquired with a tube voltage of 120 kVp, a tube current of 190 mAs, a collimation of 32 × 1.2 mm, and a pitch of 0.9. Arterial and portal venous phase images were acquired 25 and 70 seconds after the start of the injection of contrast medium. A fixed scan delay was used for the arterial phase. The dual-energy mode was used for both arterial and portal venous phase imaging, with tube voltages of 100 kVp and 140 kVp with a tin filter, tube currents of 230 and 178 mAs, a collimation of 32 × 0.6 mm for both tubes, a pitch of 0.55, and a gantry rotation time of 0.5s. Non-ionic contrast medium (Iohexol, 300 mg/dL; GE Healthcare, USA) was injected intravenously at a flow rate of 3 mL/s. The amount of contrast medium injected was calculated according to the patient weight (2 mL/kg).

### Image evaluation

To determine the tumor T stage using conventional CT signs, and to compare the sensitivity and specificity between un-enhanced CT and dual-energy CT two experienced abdominal radiologists evaluated the three-phase images by consensus in a joint session. The definition of stages T1–T4 followed the 7^th^ edition of the staging manual published by the American Joint Committee on Cancer in 2010 [11].

The pre-contrast, arterial and venous phase images were reconstructed with a 1.5-mm slice thickness and a B30 kernel. The arterial and venous phase images were obtained by mixing high- and low-energy images in a 1:1 ratio, which was the default mixing ratio. These mixed images were considered as simulated single energy 120 kVp images. Image reading was performed on a commercial workstation (MMWP; Siemens Healthcare, Germany) using transverse, MPR or maximum intensity projection views. Both readers were blinded to the results of the DECT iodine measurements and histologic investigations.

**Fig 1:**
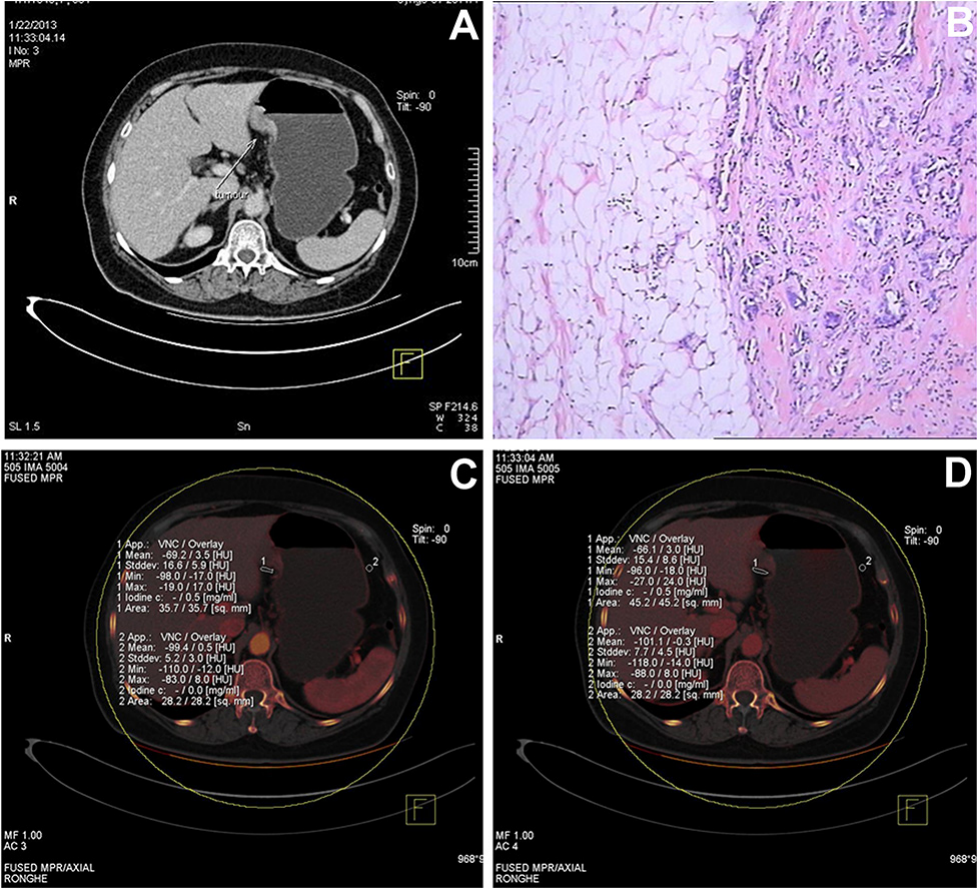
Representative CT images obtained from a 68 year-old female patient. (A) The mixed venous phase image shows thickening of the wall of the lesser curvature with transmural enhancement (arrow). Based on the mixed image, the tumor was classified as stage cT3. (B) The histological image, stained with hematoxylin and eosin (HE, ×100), revealed a grade II adenocarcinoma with invasion of surrounding soft tissue. The pathologic stage was pT4a. (C) The iodine map image at the arterial phase shows that the iodine concentration was 0.5 mg/mL in the fat near the tumor (ROI 1), but 0 mg/mL in the fat in a normal region distant from the tumor (ROI 2). (D) The iodine map image at the venous phase shows that the iodine concentration was 0.5 mg/mL in the fat near the tumor (ROI 1), but 0 mg/mL in the fat in a normal region distant from the tumor (ROI 2). This indicates that the serosa was invaded by the tumor.

To prepare the image for iodine quantification, high- and low-energy arterial and venous phase images were reconstructed with a 5-mm slice thickness and a D30 kernel. The iodine concentration was determined by one radiologist using a commercial dual-energy software package (Liver VNC; Siemens Healthcare, Germany). The iodine concentration was measured by selecting a region of interest (ROI) in the perigastric fat adjacent to the tumor (Fig 1). A strip ROI of 25–50 mm^2^ and a width ≤5 mm (considering the range of cancer invasions, we limited the width of the ROI) of the cancer tissue was selected close to and along the gastric wall (we kept a 1 mm gap between the cancer tissue and gastric wall, so the latter was not involved) to measure the adipose iodine concentration in the involved gastric serosa. To obtain a control value for the iodine concentration in fat, an additional ROI was placed in an area distant to the tumor, for example at the greater curvature (Fig 1C and 1D). The ROI was 25–50 mm^2^ circular and positioned so that it was over a homogenous area, and did not overlap with regions containing tumor or other tissues such as blood vessels. Each measurement was repeated 3 times, and the average iodine concentration recorded for further analysis. In each patient, the iodine concentration was measured from both arterial and venous phase images, using a ROI of the same size, shape and positioned at the same anatomic location. Only the means were used for statistical analyses. Since gastric peristalses lasted throughout the whole process and the ROIs were selected manually, we could not guarantee that the ROI selections during the arterial and venous phases were exactly the same. Therefore, we tried our best to ensure a similar choice in shape, size and site.

### Histologic examination of resected tumor

All specimens obtained by surgery were embedded in paraffin, stained with hematoxylin and eosin (HE) using standard techniques, and then sectioned into slices 4 μm thick. Light microscopy was used to determine the pathologic type, histologic grade and invasion depth of the tumor, and the presence/absence of lymph node metastasis.

### Radiation dose

The volume CT dose index (CTDIvol) and dose length product (DLP) were recorded from the CT console for the pre-contrast, arterial and venous phase scans. The effective dose was calculated by multiplying the DLP by a conversion coefficient for the abdomen (k = 0.015 mSv·mGy^-1^·cm^-1^).

### Statistical analysis

Statistical analysis was performed using SPSS version 11.5 (SPSS Inc., USA). Comparisons of the mean iodine concentration between groups A and B were made using the Wilcoxon signed rank test (the data were not normally distributed). A p-value < 0.05 was considered to be statistically significant. Receiver operating characteristic (ROC) curve analysis was used to determine the utility of perigastric fat iodine concentration for diagnosing T4-stage gastric cancer. The area under the ROC curve (AUC) was used to determine the optimal threshold iodine concentration for tumor classification. Sensitivity was calculated as the true positive rate (number of true positives divided by the sum of the number of true positives and number of false negatives); specificity as the true negative rate (number of true negatives divided by the sum of the number of true negatives and number of false positives); and accuracy as the sum of the number of true positives and true negatives, divided by the total number of positives and negatives.

## Results

### Patient demographic and clinical characteristics

Of the 80 patients initially screened for inclusion in the study, 21 were excluded because surgery was not performed within one week of imaging and pathology results were not available as a reference. Of these, 5 were unresectable and received chemotherapy, 2 refused any therapy and 14 accepted neoadjuvant chemotherapy. Neoadjuvant chemotherapy is recommended for stage T4 tumors in our hospital so those patients who were included with confirmed T4 stage tumors had opted according to their own judgement to receive early surgery. 5 patients with fat layers that were too thin for measurement by CT, considered to have technique failure, were included in the calculation for sensitivity and specificity but not in the ROC curve analysis. The patients included 1 with T1, 1 with T3 and 3 with T4 stage tumors. Hence, a total of 54 patients (41 males, 13 females; mean age, 61.6 ± 10.5 years; age range, 31–78 years) were included in all of the analysis (Table 1) and 59 were included in the sensitivity and specificity analysis. There were no significant differences between groups A and B in terms of age, gender and location of the gastric carcinoma, but there were significant differences in the surgical treatment (p = 0.011) and the pathologic type of carcinoma (p = 0.010). The gastric carcinoma was located in the gastric cardia in 10 patients, cardia-fundus in 11, corpus in 16 and antrum in 17. All patients were treated surgically by radical total gastrectomy (15 patients) or radical subtotal gastrectomy (39 patients). All patients received D2 lymph node dissection. The pathologic types of gastric cancer identified in these patients included adenocarcinoma in 46 patients (G1, well differentiated in 3; G2, moderately differentiated in 26; and G3, poorly differentiated in 17), mucinous adenocarcinoma in 3 patients, signet ring cell carcinoma (SRCC) in 4 patients and adenocarcinoma combined with mucinous adenocarcinoma in 1 patient.

**Table 1:** Clinical and demographic characteristics of the 54 patients.

Characteristic	Total	Group A	Group B	p value (Group A Vs. Group B)
Gender (no. of patients)				
Male	41	23	18	0.401
Female	13	9	4	
Age (years)				
Mean ± standard deviation	61.6 ± 10.5	62.5±11.2	60.3±10.1	0.071
Anatomic location of gastric carcinoma (no. of patients)				
Antrum	17	10	7	0.993
Corpus	16	9	7	
Cardia-fundus	11	6	5	
Cardia	10	6	4	
Surgical treatment (no. of patients)				
Radical subtotal gastrectomy	39	19	20	0.011
Radical total gastrectomy	15	13	2	
Pathologic type (no. of patients)				
Adenocarcinoma	46	24	22	0.010
well differentiated	3	0	3	
moderately differentiated	26	11	15	
poorly differentiated	17	13	4	
Signet ring cell carcinoma	4	4	0	
Mucinous adenocarcinoma	3	3	0	
Adenocarcinoma combined with mucinous adenocarcinoma	1	1	0	

### Tumor staging based on pathology of resected specimens

TNM staging, based on post-surgical pathology results, was as follows: 8 patients were classified as T1, 7 as T2, 7 as T3 and 32 as T4; 24, as N0, 16 as N1, 6 as N2 and 8 as N3; no patients had distant metastasis. Seven patients were classified as stage IA, 7 as IB, 6 as IIA, 8 as IIB, 14 as IIIA, 4 as IIIB 4, and 8 as IIIC. According to the T-stages, 32 patients were assigned to Group A, and 22 to group B.

### Tumor staging based on conventional CT imaging

**Fig 2:**
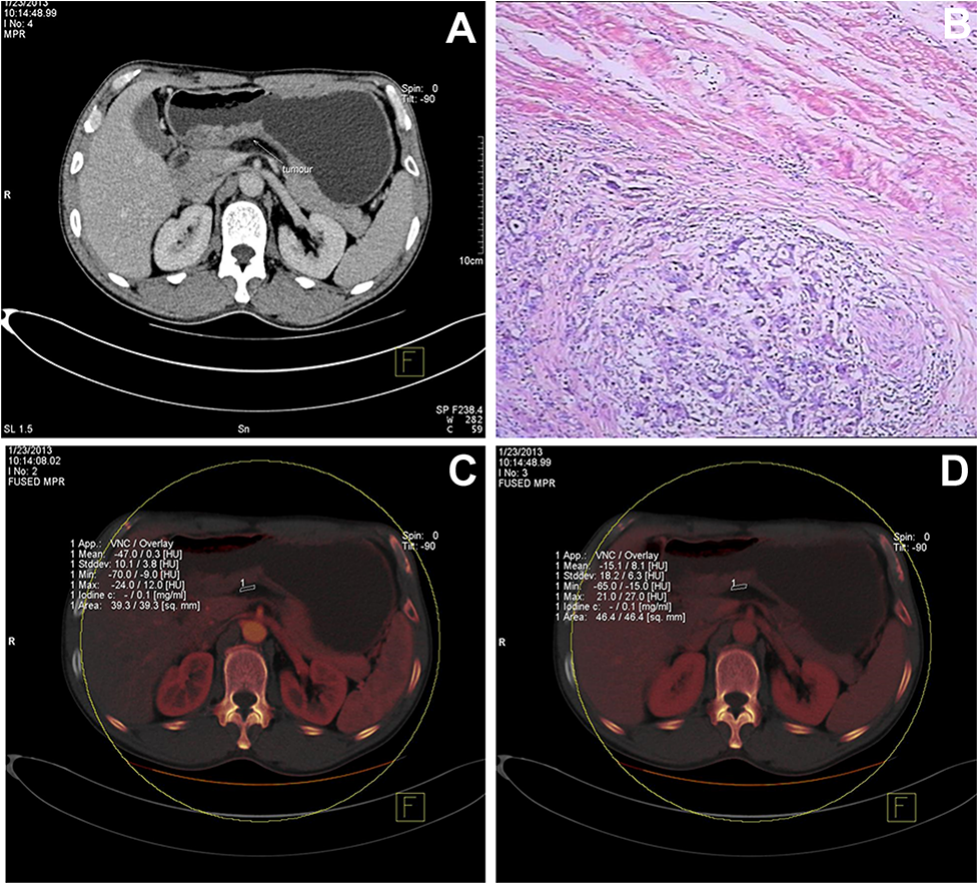
Representative CT images obtained from a 46 year-old male patient. (A) The mixed venous phase image depicts thickening of the wall of the antrum with transmural enhancement. The density of the perigastric fat was elevated, with a stripe-like shadow. The clinical stage was determined to be cT4. (B) The histological image, stained with hematoxylin and eosin (HE, ×100), revealed a grade II adenocarcinoma with muscularis invasion. The pathologic stage was pT3. (C) The iodine map at the arterial phase shows that the iodine concentration was 0.1 mg/mL in the perigastric fat (ROI 1). (D) The iodine map at the venous phase shows that the iodine concentration was 0.1 mg/mL in the perigastric fat (ROI 1).

Based on images acquired using conventional CT scanning, 3 patients were classified as having cancer of stage T1, 10 as T2, 18 as T3 and 23 as T4 (Table 2, Figs 1 and 2, S1 and S2 Figs). Compared with histologic staging, 22 patients were incorrectly classified into other T stages by conventional CT, including 4 cases of pathologic T3 misclassified by CT as T4, and 13 cases of pathologic T4 misclassified by CT as T3 or T2. Using the histologic results as the reference, the T stage was correctly identified in 57.6% (34/59) by evaluation of conventional CT images. Furthermore, the accuracy of conventional CT for distinguishing stage T4 from non-T4 stages was 67.8% (40/59).

**Table 2:** T-staging of the gastric cancers using preoperative conventional pre-contrast single-energy CT and dual-energy CT (DECT) compared to postoperative histology staging.

		CT stage		DECT stage		Total
		Non T4	T4	Non T4	T4	
**Arterial phase**						
Histologic stage	Non T4	18	4^a^	19	3^a^	22
	T4	13^b^	19	5^b^	27	32
Total		31	23	24	30	
**Venous phase**						
Histologic stage	Non T4	18	4^c^	19	3^c^	22
	T4	13^d^	19	4^d^	28	32
Total		31	23	23	31	

The data represents patient numbers ^a^ In the arterial phase 3 patients with non T4 histologic stage was wrongly diagnosed as T4 by conventional CT staging that was corrected by DECT.

^b^ In the arterial phase 10 patients were correctly diagnosed by DECT after a wrong diagnosis by conventional CT.

^c^ In the venous phase 2 patients with non T4 histologic stage was wrongly diagnosed as T4 by conventional CT staging that was corrected by DECT.

^d^ In the venous phase there were 12 patients correctly diagnosed by DECT after a wrong diagnosis by conventional CT.

### Tumor staging based on DECT measurements of iodine concentration

As shown in Table 3, the iodine concentration of the fat adjacent to the tumor was significantly higher in group A than in group B for both arterial phase images (0.60 ± 0.34 mg/mL [range, 0.00–1.30 mg/mL] *vs*. 0.09 ± 0.19 mg/mL [range, 0.00–0.80 mg/mL]; p < 0.001) and venous phase images (0.83 ± 0.41 mg/mL [range, 0.00–1.80 mg/mL] *vs*. 0.27 ± 0.21 mg/mL [range, 0.00–0.90 mg/mL]; p < 0.001). In contrast, there were no significant differences between groups A and B in the iodine concentration of fat at sites distant from the tumor for both the arterial phase (0.02 ± 0.07 *vs*. 0.02 ± 0.04) and venous phase (0.12 ± 0.20 *vs*. 0.04 ± 0.09). In group A, there was a significant difference in iodine concentration between fat adjacent to the tumor and that at distant sites, both for the arterial and venous phases (p < 0.001). In group B, there was also a significant difference in iodine concentration between fat adjacent to and that distant from the tumor for the venous phase (p < 0.001), but not for the arterial phase (p = 0.12).

**Table 3:** Iodine concentrations in the perigastric adipose tissue of patients in groups A and B, measured using DECT.

	Group A (n = 32)		Group B (n = 22)	
	Near tumor	Distant to tumor	Near tumor	Distant to tumor
Arterial phase	0.60 ± 0.34	0.02 ± 0.07^§^	0.09 ± 0.19*	0.02 ± 0.04
Venous phase	0.83 ± 0.41	0.12 ± 0.20^§^	0.27 ± 0.21*	0.04 ± 0.09^§^

Data are presented as the mean ± SD (mg/mL). Near tumor: iodine concentration measured in a ROI near the tumor; Distant to tumor: iodine concentration measured in a ROI distant to the tumor.

*p < 0.001 compared with the corresponding value in group A

^§^p < 0.001 compared with the ‘Near tumor’ value for the same phase within the same group.

### ROC curve analysis

ROC curve analysis of using DECT-derived measurements of perigastric fat iodine concentration to stage gastric cancer showed that the AUC was 0.89 for arterial phase images and 0.90 for portal venous phase images. For arterial phase images, the optimal threshold iodine concentration (in a ROI near the tumor) for distinguishing between group A and B was 0.25 mg/mL, and this yielded a sensitivity of 77.1%, a specificity of 79.2%, and an accuracy of 78.0%. For venous phase images, the optimal threshold value was 0.45 mg/mL, and its use resulted in a sensitivity of 80.0%, a specificity of 79.2%, and an accuracy of 79.7%. By not considering the 5 (8.5%) patients with technique failure, the sensitivity and specificity can be improved to 84.4%, 86.4% for arterial phase images, and 87.5%, 86.4% for venous phase images, as the cutoff value was actually calculated without these cases. Even with technique failure the method is still viable for over 90% of patients.

### Radiation dose

The CTDIvol, DLP and effective dose were (respectively) 14.00 ± 0.41 mGy, 338.60 ± 80.14 mGy-cm and 5.08 **±** 1.20 mSv for the pre-contrast phase; 12.66 ± 2.90 mGy, 292.87 ± 97.10 mGy-cm and 4.39 **±** 1.46 mSv for the arterial phase; and 12.58 ± 2.70 mGy, 305.10 ± 161.60 mGy-cm and 4.58 **±** 2.42 mSv for the venous phase.

## Discussion

The present study was designed to investigate the feasibility of using the iodine concentration in perigastric adipose tissue adjacent to the tumor, measured with DECT, to detect serosal invasion of gastric cancer. The main findings of the study were that the iodine concentration in perigastric adipose tissue adjacent to the tumor was significantly higher in the presence of serosal invasion than when the serosa was intact. Using post-surgery histologic findings as a ‘gold standard’ for staging, ROC curve analysis revealed that the AUC for detecting serosal invasion was 0.89 and 0.90 for arterial and portal venous phases, respectively. When 0.25 and 0.45 mg/mL were taken as threshold iodine concentration value for the arterial and portal venous phases, respectively, the accuracy of DECT for differentiating between T4a-stage and earlier T-stage gastric cancer was 78.0% and 79.7%, respectively. Taken together, these observations demonstrate that quantification of iodine in perigastric adipose tissue by DECT represents a novel and accurate clinical method for distinguishing T4a-stage gastric cancer from earlier T-stages. To the best of our knowledge, this is the first report demonstrating the utility of this approach in the staging of gastric cancer.

DECT iodine measurements provide a quantitative imaging method for detecting advanced local gastric cancer. DECT has been used to characterize various tumors, such as lung cancer nodules [12], insulinoma [13] and adrenal nodules [14]. The present study extended the scope of DECT to advanced gastric cancer. It was found that invasion of the serosa by gastric cancer significantly elevated the iodine concentration in the perigastric adipose tissue adjacent to the tumor. In contrast, adipose tissues without tumor invasion and a normal blood supply showed undetectable or low iodine levels in the arterial and portal venous phases. The high iodine concentration observed in the perigastric adipose tissue of patients with T4a-stage cancer is likely associated with increased perfusion, possibly caused by tumor invasion or leakage from malignant cell membranes as a result of a breakdown in serosal integrity.

As evidenced by the findings in the present study, DECT provides additional value to conventional single-energy CT in the diagnosis of T4a-stage gastric cancer. According to the national comprehensive cancer network (NCCN) clinical practice guidelines for gastric carcinoma 2010 (Chinese version), preoperative chemotherapy or chemoradiotherapy can be considered in resectable advanced tumors or those with node metastasis. As it is difficult to make a confirmed diagnosis in some patients with conventional CT techniques, iodine concentration imaging may assist with the decision on whether to undertake preoperative adjuvant therapy. When equipped with sub-millimeter thin slicing, single-energy CT can readily depict the serosa adjacent to the epigastric fat [5,14], and the accuracy of diagnosing T4 gastric cancer is significantly improved with the assistance of the MPR technique [5,7,15–17] or virtual gastroscopy [18]. As the gastric serosa is very thin, we could not observe serosal invasion directly. Conventional MDCT determines whether the gastric serosa is invaded mainly by estimating the density of adipose tissues at the serosal surface by direct observation; however, the serosal surface is very rough and the adjacent adipose tissues are generally turbid, thus increased density could reflect several different phenomenon including tumor invasion and reactive fibrous connective tissue hyperplasia. Therefore, the specificity of determining serosal invasion by MDCT is relatively low. In addition, for T4a lesions with perigastric microinvasion, even increasing the window width and window level in conventional MDCT could not clearly determine serosal invasion. In the present study, all the patients were treated surgically, and preoperative staging of the lesions was mainly non-T4; however, according to the postoperative pathological results, 12 patients had T4a lesions but were underestimated as T3, and 1 patient had a T4a lesion but was underestimated as T2. These findings demonstrated the limitations of using MDCT in evaluating serosal invasion. The results of the present study demonstrate that DECT iodine quantification represents an accurate method with which to identify T4a-stage gastric cancer, which is comparable in accuracy, sensitivity and specificity to previous studies using MPR images [5,7,8,15]. However, identification of all T4a patients by DECT alone was still not possible. We tested higher sensitivity values of 95% and 100% for the arterial and venous phase, but the resulting specificities were too low to suggest a viable clinical use for the DECT technique in isolation. Therefore, this technique may be useful in addition to other methods or further research may identify methods by which the sensitivity and specificity of the DECT technique can be improved. The iodine maps provided by DECT are color maps, which are better for the color distinguishing ability of humans compared with grayscale images provided by conventional MDCT. In addition, iodine concentration provided by DECT is quantitative data, which could provide better objective evidence for the diagnosis. Abnormal tumor angiogenesis and local microcirculation (compared with normal tissues) exist at the regions with cancerous cell invasion at early stages. DECT could evaluate the microcirculation of the region of interest by measuring the iodine concentration at the serosal adipose tissues, and thus help determine serosal invasion. Therefore, the findings of the present study demonstrated that using DECT to measure iodine concentration could provide more objective and accurate evidence for determining serosal invasion. In addition to enabling iodine quantification, DECT can improve the visualization of invading tumor, adjacent structures and neighboring blood vessels through the use of monochromatic MPR images [13,19], such as those shown in Figs 1C and 2C, without a radiation dose penalty [20]. Therefore, an advantage of DECT is that it allows the evaluation of linear or reticular fat stranding signs as well as iodine concentration, which may be useful in cases of inflammatory reactions.

The accuracy of DECT in this study was slightly lower than those studies that used MDCT, which were estimated to be nearly 90% [5,7,9]. However, there are many points to consider when comparing the two methods. The first is the slight alteration in the staging criteria. The 7^th^ TNM staging criteria used here [3], in 2010 increased the requirement of the display of different layers of the gastric wall, and thus increased the difficulty of accurate staging by preoperative CT scanning. For instance, tumors with muscular and subserosal invasions were classified as stage T2 in the 6th criteria, while in the 7th criteria, tumors with muscular invasion were classified as stage T2, while tumors with subserosal invasion were classified as stage T3; tumors with serosal invasion were classified as stage T3 or T4 in the 6th criteria but as stage T4 in the 7th criteria. The use of different staging criteria could decrease the comparability of the studies. In addition, the different data of these studies could be associated with the differences in the scanning equipment, examination method (e.g. gastrointestinal preparation before the examination), clinicians’ experience, and different patient subgroups included. In the present study, the density of serosal adipose tissues was evaluated by the naked eye (similar to MDCT), and the accuracy of determining serosal invasion was 68.5%; while for preoperative examinations, iodine concentration was measured by DECT to evaluate serosal invasion, and the accuracy was 78.0% (arterial phase) and 79.7% (venous phase), respectively, compared with the gold standard (pathological examinations).

The present study has some limitations. First, only patients with confirmed gastric cancer were enrolled in our study, which may overestimate the capability of DECT for staging gastric cancer. Second, the number of patients included was relatively small, so it should be considered to be a pilot study that merits larger-scale studies to confirm the results. Third, direct comparisons with other imaging modalities were not made. Fourth, the utility of iodine quantification with DECT was examined only for the diagnosis of T4-stage gastric cancer; the ability of DECT to diagnose earlier stages was not examined and there may be some other factors that will affect the iodine concentration measurement such as inflammation, metastatic lymph nodes, or perigastric tumor deposits close to the tumor. So the value of this method will have to be evaluated in further studies involving all of these factors. Finally, some patients were excluded from the study due to insufficient perigastric fat for measurement of iodine concentration using DECT–thus, this methodology may not be appropriate for all patients, and this may have introduced some bias into the study as these patients were not included in the sensitivity and specificity calculations.

## Conclusions

Quantification of iodine content in perigastric adipose tissue with DECT provides an accurate, sensitive and specific method for distinguishing gastric cancer with serosal invasion from that without serosal invasion. Thus, DECT represents a useful clinical tool for preoperatively diagnosing T4a-stage gastric cancer.

## Supporting Information

S1 Data Raw data. (XLSX)

S1 Fig Representative CT images obtained from a 63 year-old male patient. **A:** Venous phase: cardia wall thickening, with a nontransmural enhanced wall (arrow), preoperative imaging staging: T1. **B:** Postoperative pathological images, (HE, X40), showed a low differentiated adenocarcinoma that had infiltrated the mucous layer. Postoperative pathologic staging: pT1. **C**: arterial phase IC = 0.0 mg/ml. **D**: venous phase IC = 0.0 mg/ml, indicated no serous invasion.
(PDF)

S2 Fig Representative CT images obtained from a 50 year-old female patient. **A:** Venous phase: cardia wall thickening, with a nontransmural enhanced wall (arrow), preoperative imaging staging: T2. **B:** Postoperative pathological images, (HE, X200), showed a low differentiated adenocarcinoma that had infiltrated the muscle layer. Postoperative pathologic staging: pT2. **C**: arterial phase IC = 0.0 mg/ml. **D**: venous phase IC = 0.0 mg/ml, indicated no serous invasion.
(PDF)
