# Supplementary figures and images for: Quantification of the Iodine Content of Perigastric Adipose Tissue by Dual-Energy CT: A Novel Method for Preoperative Diagnosis of T4-Stage Gastric Cancer
Source: PLoS One. 2015 Sep 15;10(9):e0136871. doi: 10.1371/journal.pone.0136871 (PMC4570799; doi:10.1371/journal.pone.0136871)

**A**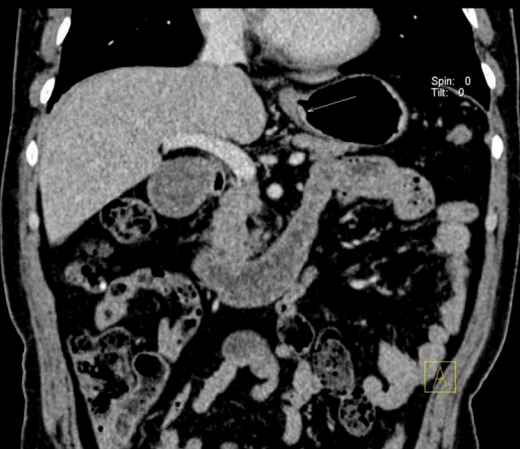**B**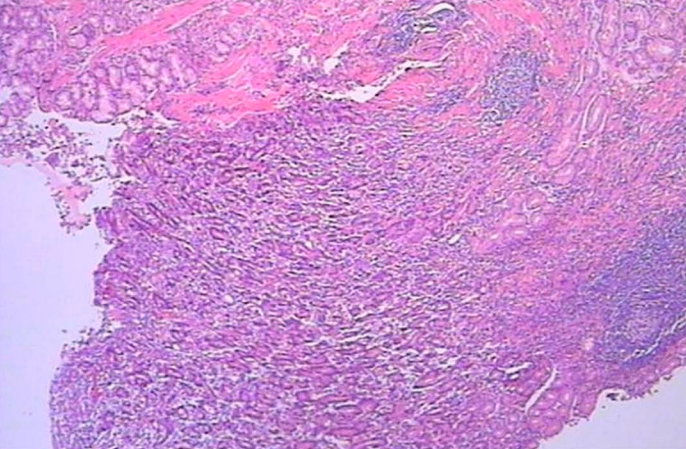**C**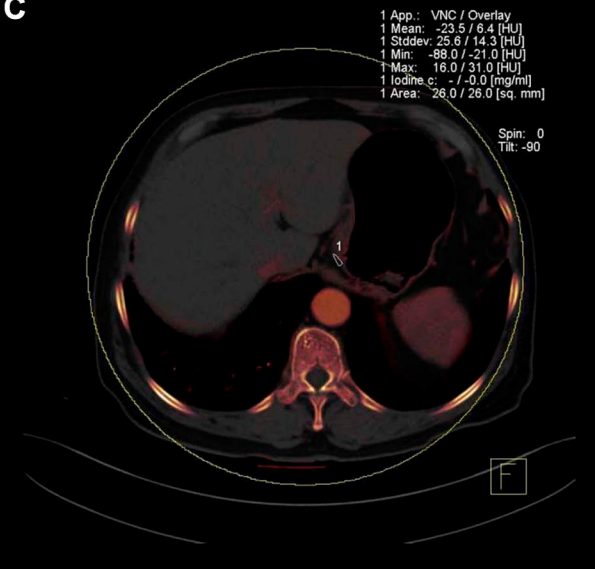**D**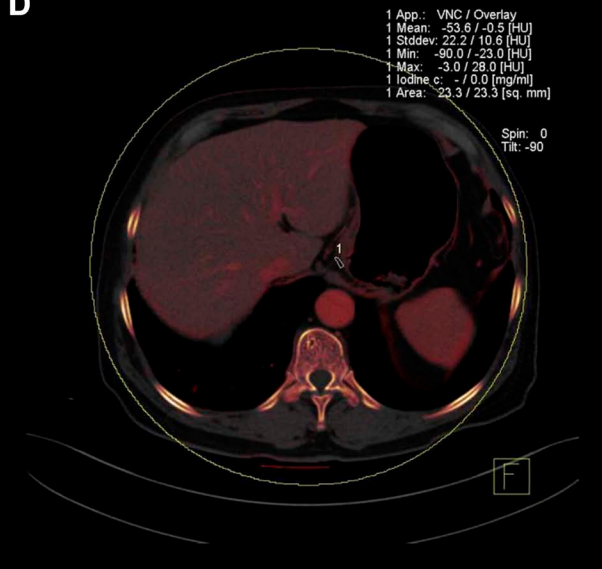

Supplement: S1 Fig — A: Venous phase: cardia wall thickening, with a nontransmural enhanced wall (arrow), preoperative imaging staging: T1. B: Postoperative pathological images, (HE, X40), showed a low differentiated adenocarcinoma that had infiltrated the mucous layer. Postoperative pathologic staging: pT1. C: arterial phase IC = 0.0 mg/ml. D: venous phase IC = 0.0 mg/ml, indicated no serous invasion. (PDF) [file pone.0136871.s002.pdf]

**A**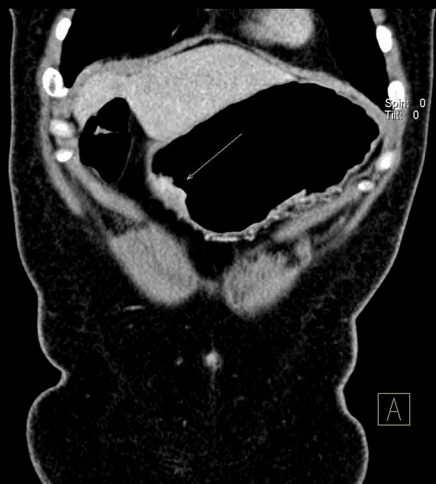**B**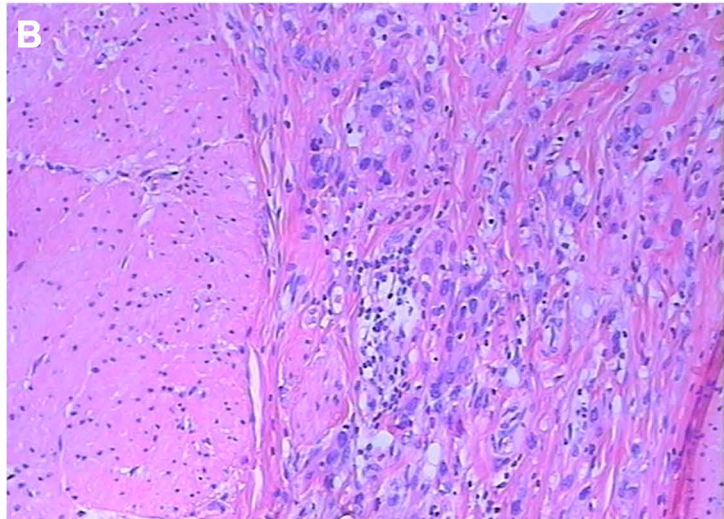**C**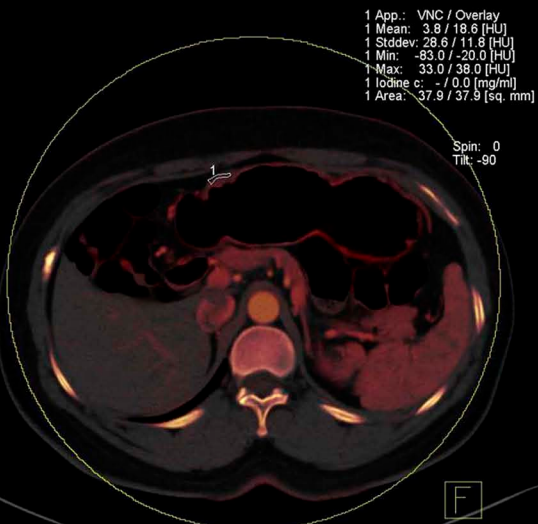**D**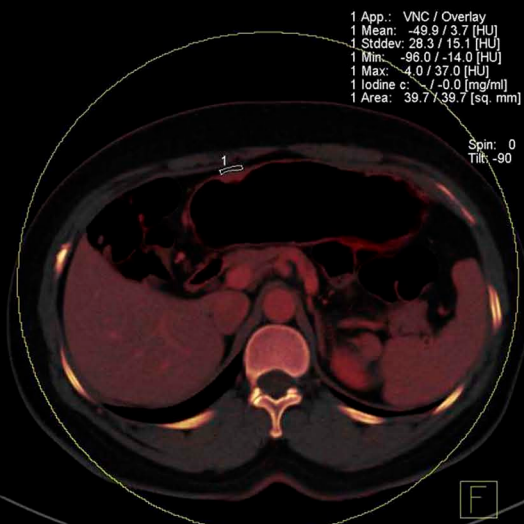

Supplement: S2 Fig — A: Venous phase: cardia wall thickening, with a nontransmural enhanced wall (arrow), preoperative imaging staging: T2. B: Postoperative pathological images, (HE, X200), showed a low differentiated adenocarcinoma that had infiltrated the muscle layer. Postoperative pathologic staging: pT2. C: arterial phase IC = 0.0 mg/ml. D: venous phase IC = 0.0 mg/ml, indicated no serous invasion. (PDF) [file pone.0136871.s003.pdf]
